# Psychotropic drugs intake in people aging with intellectual disability: Prevalence and predictors

**DOI:** 10.1111/jar.12996

**Published:** 2022-04-05

**Authors:** Laura García‐Domínguez, Patricia Navas, Miguel Ángel Verdugo, Víctor B. Arias, Laura E. Gómez

**Affiliations:** ^1^ Institute on Community Integration University of Salamanca Salamanca Spain; ^2^ University of Oviedo Oviedo Spain

**Keywords:** aging, behavioural problems, intellectual disability, mental health, psychotropic medication

## Abstract

**Background:**

Psychotropic medication is frequently administered to people with intellectual disability with mental health and/or behavioural problems, instead of other non‐pharmacological interventions. This study describes the mental health and behavioural problems of people aging with intellectual disability, their psychotropic medication intake, and the factors contributing to a greater medication intake.

**Method:**

The sample consisted of 991 people with intellectual disability over 45 years. Descriptive statistics and multinominal logistic regression were carried out.

**Results:**

Antipsychotics were the most used psychotropic drug. Older people with mild intellectual disability living in institutions and affected by mental health and behavioural problems were more likely to take larger amounts of psychotropic medication.

**Conclusions:**

Antipsychotics continue to be widely used by people with intellectual disability and mental and behavioural health problems, especially those in institutionalised settings. Future research should consider if medication intake could be reduced providing better supports in the community and non‐pharmacological interventions.

## 1 INTRODUCTION

Psychotropic medication is frequently used to treat people with intellectual disability who exhibit mental health and behavioural problems instead of considering other nonpharmacological interventions such as behavioural therapy and positive behavioural support (Tan et al., 2015). This results in the use of high doses of medication (Bowring et al., 2017), especially antipsychotic drugs (Tsiouris, 2010), that are not always necessary. In fact, the proportion of people with intellectual disability who are prescribed psychiatric drugs is larger than that of people with intellectual disability who have a diagnosis of mental illness (Aguilar, 2019; Lunsky et al., 2018; Sheehan et al., 2015; Tsiouris et al., 2013).

The research carried out by Folch et al. (2019) reports that people with intellectual disability take psychotropic drugs even when many of them do not have a clinical diagnosis of mental illness. The study by Sheehan et al. (2015) reflects a similar result: 71% of the people with intellectual disability who use antipsychotics have no record of mental illness in their history. This leads us to consider their use as a mean of ‘restraining’ any problematic behaviour that may occur, which is consistent with previous literature (Bowring et al., 2017; de Kuijper et al., 2010; Deb et al., 2015; Gothelf et al., 2008; Scheifes, Egberts, et al., 2016; Sheehan et al., 2015).

In this regard, there is evidence that the presence of behavioural problems is associated with the start and maintenance of psychotropic drug therapy (Song et al., 2020), which is often chosen over other less invasive alternatives (i.e., psychological therapy; Deb et al., 2009; Valdovinos et al., 2017). Pharmacological interventions are commonly used to manage behavioural problems in spite of its numerous side effects (Sheehan, 2018) and the lack of awareness about the safety of psychotropic medications in population with intellectual disability (Gómez, Navas, Verdugo, & Tassé, 2021). These adverse effects may hinder the diagnosis of other health problems in the population with intellectual disability (Axmon, Ahlström, et al., 2019).

Besides behavioural problems, there are several factors that, according to the scientific literature, contribute to a greater intake of psychotropic drugs among people with intellectual disability. Living environment as a factor that may explain a greater or lesser use of psychiatric medication has also been the object of study, although results are still contradictory. While certain authors report that the use of psychotropic medication is greater among people with intellectual disability who live in community settings (Spreat et al., 2004), others claim that segregated contexts are associated with a greater use of psychiatric medication (Lunsky & Modi, 2018; O'Dwyer et al., 2016).

Increased levels of psychotropic medication prescription have also been observed in individuals with intellectual and developmental disabilities who require more extensive supports (Axmon, El Mrayyan, et al., 2019), since they may present more mental health issues requiring psychopharmacological treatment (Navas et al., 2017). Nevertheless, it is important to bear in mind that behavioural problems are attempts at communication by individuals who may lack the capacity to express themselves otherwise (Gómez & Navas, 2021) and that their assessment in people with more severe disabilities might be difficult due to their communication difficulties (Tassé et al., 2017). This could lead professionals to mistakenly consider these behavioural manifestations to be symptoms of a mental health disorder rather than responses to contextual changes or specific social situations.

Older age also appears to be positively associated with the intake of psychotropic medication (Axmon, Ahlström, et al., 2019; Bowring et al., 2017). In aging people with intellectual disability, polypharmacy and its associated risks to their health are far greater (Axmon et al., 2017; El Mrayyan et al., 2019; O'Dwyer et al., 2018), so the need to prescribe psychotropic drugs should be carefully assessed (O'Dwyer et al., 2018).

Adulthood and old age are stages of the life cycle that involve major changes and transitions. Already 20 years ago, the World Health Organization warned about the need to improve our knowledge and sensitivity as regards stressors that are associated with the general process of aging and could negatively affect the emotional wellbeing of people with intellectual disability (Hogg et al., 2000). Even though we are currently aware of the impact of transitions such as retirement (Amado et al., 2013) or stressors such as the grieving process (Dodd et al., 2005) on aging people with intellectual disability, the behavioural expressions that are triggered by them still drive professionals to overprescribe psychotropic medication (Axmon et al., 2017; Eady et al., 2015). Research on the prescription of psychotropic medication among this population group is, however, limited (Chitty et al., 2016; O'Dwyer et al., 2017), and stronger scientific evidence concerning the use and treatment with psychotropic drugs in aging people with intellectual disability is required (Sheehan et al., 2015).

For the reasons mentioned above, the aims of this study focus on: (a) describing the mental health and behavioural problems presented by aging people with intellectual disability, as well as the type of psychotropic medication they use; and (b) analysing the factors that predict a greater intake of psychiatric drugs in older people with intellectual disability.

## 2 METHODS

### 2.1 Participants

The sample consisted of 991 adults with administratively defined intellectual disability (i.e., receiving services aimed at population with intellectual disability) aged between 45 and 88 (*M* = 55.3; *SD* = 7.3). Table 1 gathers the sociodemographic data of the study participants.

**TABLE 1:** Sociodemographic data of people with intellectual disabilities participating in the study

Sociodemographic data of participants		
Gender	*N*	%
Male	488	49.2
Female	503	50.8
*Severity of intellectual disability*		
Mild	253	25.5
Moderate	420	42.4
Severe/profound	318	32.1
*Living environment*		
Residential setting	525	53.0
Community setting	459	46.3
Unknown	7	0.7
*Communication type*		
Verbal	869	87.7
Non‐verbal	110	11.1
None	10	1.0
Unknown	2	0.2

A total of 353 informants participated in the study by completing a survey, which will be subsequently detailed, on the health status of the person with intellectual disability. Most of them were females (75.4%) and the average age was 50.2 years old (*SD* = 15.8). More than half were professionals (58.9%) and almost 40% were family members. All of them had known the person assessed for at least 12 months. Most of them (74.0%) had daily contact with the person with intellectual disability.

### 2.2 Instrument

An ad hoc questionnaire was prepared to obtain information about the health status of aging people with intellectual disability. Its details and development process can be consulted in the article by García‐Domínguez et al. (2020). This study focuses on the analysis of the findings in the following sections of the instrument:

1. *Mental health conditions*: this section assessed the presence/absence in the person of mental health problems listed in the Diagnostic and Statistical Manual of Mental Disorders (DSM‐5; American Psychiatric Association, 2013), which are presented in Table 2. The respondents had to identify their presence when there was a clinical diagnosis issued by a professional.
2. *Behavioural problems*: in this section, the behavioural problems that the person assessed had exhibited in the last 12 months were evaluated. A list with a brief description of behavioural problems was included (e.g., ‘inappropriate touching of others’ was an example for socially offensive behaviour) according to the categories used in the Inventory for Client and Agency Planning (ICAP; Bruininks et al., 1986) and the Behaviour Problems Inventory‐ Short Form (BPI‐S; Rojahn et al., 2012): stereotyped behaviour, self‐injurious behaviour, aggressive/destructive behaviour, socially offensive behaviour, disruptive behaviour, other.
3. *Medication use*: in this section, the respondents were asked to detail the psychotropic drugs (indicating full name and dosage) taken by the person assessed during the 2 months prior to the administration of the questionnaire.

**TABLE 2:** Prevalence of mental health conditions and behavioural problems

Mental health conditions^a^	Yes		No	
	*N*	%	*N*	%
Destructive disorders	162	16.5	818	83.5
Anxiety disorders	150	15.5	820	84.5
Depressive disorders	146	15.1	822	84.9
Schizophrenia spectrum disorders	99	10.1	882	89.9
Obsessive–compulsive disorders	84	8.6	892	91.4
Sleeping disorders	75	7.6	906	92.4
Personality disorders	42	4.3	943	95.7
Dementia/AD	42	4.3	936	95.7
Bipolar disorders	34	3.5	947	96.5
Eating disorders	34	3.4	953	96.6
Excretion disorders	28	2.8	961	97.2
Addictive disorders	13	1.3	975	98.7
Dissociative disorders	9	0.9	969	99.1
Other mental health condition	15	1.6	942	98.4
Behavioural problems^b^	Yes		No	
	*N*	%	*N*	%
Stereotyped behaviour	259	26.9	703	73.1
Aggressive behaviour	240	25.3	710	74.7
Disruptive behaviour	202	21.1	756	78.9
Socially offensive behaviour	105	11.0	849	89.0
Self‐injury behaviour	81	8.4	878	91.6
Other behavioural problem	40	4.3	889	95.7

a Mental health conditions listed in the Diagnostic and Statistical Manual of Mental Disorders (DSM‐5).

^b^ Behavioural problems were selected according to the categories used in the Inventory for Client and Agency Planning (ICAP) and the Behaviour Problems Inventory‐ Short Form (BPI‐S).

### 2.3 Procedure

Initial contact was established with 1068 organisations that are part of the four main service providers for people with intellectual disability in Spain (Plena inclusión, Down España, ASPACE España, Autismo España), out of which 227 (21.0%) expressed an interest in taking part in the study given that there were aging individuals among their users. The final sample was obtained from 83 of these organisations, located in 34 of the 50 Spanish provinces.

The survey used in this cross‐sectional study was sent by post or email (according to preference) to the participating organisations, together with the following two documents: (a) an informed consent form that was to be returned by the professionals or relatives before recording the data, and (b) an information letter stating the study objectives, method, funding sources and other relevant information regarding the research, as well as a contact address for further information if required. The research team maintained continuous contact with the respondents both by email and telephone to solve any questions that might arise throughout the data collection process.

Once all the surveys were received (by postal mail or e‐mail), the data were transferred to an SPSS 25 database for subsequent analysis. Informants were contacted by phone or email to request clarification of the information provided when necessary.

Regarding medication, informants provided data on the name of psychotropic drugs taken by the person, but in many cases did not specify the dosage. The research team decided to limit the analysis to the type of drug, and the first author coded this information taking into account its main use. The answers were classified by the first author into: anxiolytics, antidepressants (including mood stabilisers or euthymizers) and antipsychotics, so results could be compared with those of previous research (e.g., Lunsky & Modi, 2018; Tsiouris et al., 2013), taking also into account that these are the psychotropic drugs most widely used in the general population with intellectual disabilities (e.g., Bowring et al., 2017; Sheehan et al., 2015; Song et al., 2020).

This procedure was approved by the Bioethics Committee of the University of Salamanca. All procedures performed in this research were in accordance with the 1964 Helsinki Declaration and its later amendments or comparable ethical standards.

### 2.4 Data analysis

Descriptive statistics (frequencies and percentages) were calculated to summarise the prevalence of medication use. The association between medication use and the presence of behavioural or mental health problems was also investigated using Chi‐square test. To further study factors associated with medication use, a three‐category variable was constructed (no medication, a single psychotropic drug, and more than one psychotropic drug‐polypharmacy), and multinomial logistic regression was performed. A binary logistic regression was previously conducted with two levels in the variable (no medication and medication use). Adding a third level, thus differentiating between single drug use and polypharmacy, improved the information provided by the model. Factors that have been associated with greater medication use in previous research were included in the multivariate model (i.e., age, gender, severity of intellectual disability, communication type, living environment, mental health conditions, and behavioural problems). Information on these variables was collected through the survey previously described (sections a and b). All the variables were entered simultaneously into the model. No special treatment of missing data on the different variables was performed, since they did not account for more than 3% of the total in any case. No outliers that might significantly affect the data analysis were detected. Multicollinearity among the independent variables was tested using the variance inflation factor (VIF) with a cutoff value of <2 (John, 1983), so that a variable's VIF value being above 2 would indicate collinearity associated with such variable. All the VIF values were below 2, so multicollinearity among variables was ruled out.

The data were analysed using IBM SPSS Statistics version 25 software. The researchers set the significance level at *α* = .05.

## 3 RESULTS

### 3.1 Prevalence of mental health and behavioural issues

A total of 508 people (51.3%) exhibited mental health conditions in the 12 months prior to the application of the questionnaire. Destructive disorders were the most prevalent ones (16.5%), followed by anxiety disorders (15.5%), depressive disorders (15.1%) and schizophrenia spectrum disorders (10.1%). Behavioural problems were reported in 54.5% (*n* = 540) of the sample, the most prevalent being stereotyped behaviour (26.9%), aggressive behaviour (25.3%) and oppositional behaviour (21.1%). The frequencies of each mental health condition and behavioural problems are shown in Table 2.

### 3.2 Use of psychotropic medication

Of the entire sample, 38.4% (*n* = 381) used some type of psychotropic medication. Antipsychotics were the most common (*n* = 274; 71.9%), followed by antidepressants (*n* = 187; 49.1%) and anxiolytics (*n* = 181; 47.5%). Table 3 shows the distribution of psychotropic medication in people with mental health or behavioural problems, both types of problem, and none.

**TABLE 3:** Medication intake according to the presence of mental health conditions and behavioural problems

	Anxiolytics			Antidepressants			Antipsychotics		
	Yes *n* (%)	No *n* (%)	*χ* ^2^ (sig)	Yes *n* (%)	No *n* (%)	*χ* ^2^ (sig)	Yes *n* (%)	No *n* (%)	*χ* ^2^ (sig)
Presence of mental health problems (*N* = 140)	33 (23.6)	107 (76.4)	2.676 (.102)	37 (26.4)	103 (73.6)	5.523 (.019*)	55 (39.3)	85 (60.7)	10.369 (.001*)
Presence of behavioural problems (*N* = 172)	12 (7.0)	160 (93.0)	16.860 (.000*)	8 (4.7)	164 (95.3)	26.372 (.000*)	14 (8.1)	158 (91.9)	38.427 (.000*)
Presence of both (*N* = 368)	130 (35.3)	238 (64.7)	112.336 (.000*)	135 (36.7)	233 (63.3)	119.510 (.000*)	198 (53.8)	170 (46.2)	198.113 (.000*)
No mental health or behavioural problem (*N* = 311)	6 (1.9)	305 (98.1)	79.426 (.000*)	7 (2.3)	304 (97.7)	80.194 (.000*)	7 (2.3)	304 (97.7)	144.307 (.000*)

*Note*: *Significant differences *p* < .05.

Almost 61% of those who exhibited mental health conditions (no behavioural problems) in the past 12 months were taking some type of psychotropic medication. Antipsychotics were the most commonly used medication (*n* = 55; 39.3%). The relationship between taking antipsychotics and the presence of mental health disorders was significant (*χ* ^2^[1140] = 10.37; *p* = .001). As regards individuals with behavioural problems, the most widely used psychotropic drugs were also antipsychotics (*n* = 14; 8.1%), the relationship being statistically significant (*χ* ^2^[1172] = 38.43; *p* < .000). Over one half of the people with mental health and behavioural problems used antipsychotics (*n* = 198; 53.8%; *χ* ^2^[1368] = 198.11; *p* < .000). Of the 274 individuals who consumed antipsychotics, a total of 7 (2.3%) had no mental health or behavioural problems. Of the 172 individuals who had a behavioural problem but not a mental health condition, 22 (12.8%) were prescribed psychotropic drugs.

Table 4 shows the details of the mental health and behavioural problems exhibited by the people who consume anxiolytics, antidepressants, and antipsychotics. As might be expected, each type of psychotropic drug was taken to a larger extent by those individuals with disorders for which they are usually prescribed (e.g., 36.4% of those who used anxiolytics exhibited anxiety disorders). However, each type of psychiatric drug was also used widely by people with other conditions, this being the case with antipsychotics, whose rates of use among people affected by destructive disorders was 36.4% (*n* = 100). It is important to note that 17.7% (*n* = 32) of the people who took anxiolytics had no mental health conditions, and 21.5% (*n* = 39) did not have any known behavioural problems. Regarding antidepressants, a similar trend can be observed: 14.4% (*n* = 27) of those who used them had no mental health disorders and 24.1% (*n* = 45) had no behavioural problems. As for people taking antipsychotics, 18.2% (*n* = 50) had no mental health conditions, and 22.9% (*n* = 63) had no behavioural problems.

**TABLE 4:** Consumption of each type of psychotropic medication according to the presence of each mental health condition and behavioural problems

Mental health condition	Anxiolytics *N* = 181 (18.3%)		Antidepressants *N* = 187 (18.9%)		Antipsychotics *N* = 274 (27.6%)		None *N* = 611 (89.3%)	
	*n*	%	*n*	%	*n*	%	*n*	%
Destructive disorders	62	34.3	58	31.0	100	36.4	42	6.9
Anxiety disorders	66	36.4	56	29.9	71	25.9	38	6.2
Depressive disorders	51	28.1	81	43.3	70	25.5	39	6.3
Schizophrenia spectrum disorders	47	25.9	42	22.5	83	30.2	9	1.5
Obsessive–compulsive disorders	23	12.7	37	19.8	42	15.3	26	4.2
Sleeping disorders	32	17.6	23	12.3	38	13.8	24	3.9
Personality disorders	14	7.7	22	11.8	22	8.0	10	1.6
Dementia/AD	9	4.9	14	7.5	18	6.5	20	3.3
Bipolar disorders	16	8.8	20	10.7	27	9.8	4	.6
Eating disorders	8	4.4	13	7.0	18	6.5	9	1.5
Excretion disorders	10	5.5	5	2.7	11	4.0	10	1.6
Addictive disorders	2	1.1	4	2.1	5	1.8	6	.9
Dissociative disorders	2	1.1	4	2.1	7	2.5	1	.2
Other mental health condition	6	3.3	6	3.2	7	2.5	5	.8
No mental health condition	32	17.7	27	14.4	50	18.2	464	75.9
Behavioural problems	Anxiolytics *N* = 181 (18.3%)		Antidepressants *N* = 187 (18.9%)		Antipsychotics *N* = 274 (27.6%)		None *N* = 611 (89.3%)	
	*N*	%	*N*	%	*N*	%	*N*	%
Stereotyped behaviour	77	42.5	71	38.0	103	37.5	122	19.9
Aggressive behaviour	73	40.3	76	40.6	116	42.3	92	15.1
Disruptive behaviour	69	38.1	71	38.0	104	37.9	74	12.1
Socially offensive behaviour	37	20.4	41	21.9	57	20.8	37	6.1
Self‐injury behaviour	29	16.0	26	13.9	38	13.8	32	5.2
Other challenging behaviour	12	6.6	12	6.4	14	5.1	17	2.8
No behavioural problem	39	21.5	45	24.1	63	22.9	351	57.4

### 3.3 Factors that predict greater psychotropic medication use in older people with intellectual disability

The data yielded by the multinominal logistic regression are shown in Tables 5 and 6. Table 5 shows the results for people taking one psychotropic drug and Table 6 shows the results for people taking two or more psychotropic drugs. The results of Table 6 suggest that being older, having mild intellectual disability, living in a residential setting, having mental health conditions, and exhibiting behavioural problems contribute to increasing the probabilities of using two or more psychotropic drugs. The effect of the sex and type of communication variables on the levels of the dependent variable was not significant. Moreover, as regards polypharmacy, the results of Table 6 show how as age increases by one unit, so does the probability of using two or more drugs at a rate of 1.04 (1/.964). Having mild intellectual disability also proved a significant predictor of polypharmacy: individuals with mild intellectual disability were twice as likely to use two or more psychotropic drugs as people with severe intellectual disability (*b* = .689; *Wald χ* ^2^(1991) = 6.37; *p* = .012; *OR* = 1.99; *CI* (95%): 1.17–3.40). The probability of taking two or more psychiatric drugs was 2.76 times higher when the individual lived in a residential facility as compared to living in a community setting (*b* = 1.02; *Wald χ* ^2^(1991) = 24.58; *p* < .001). Regarding people who had some type of mental health issue, the probability of using two or more drugs proved to be 27.03 greater (1/.037) (*b* = −3.31; Wald χ^2^(1991) = 154.76; *p* < .001). Finally, another significant predictor of polypharmacy was the presence of behavioural problems (*b* = −.822; *Wald χ* ^2^(1991) = 14.54; *p* < .001), where the probability of polypharmacy use was 2.28 times higher (1/.439) when the individuals assessed had some type of problematic behaviour.

**TABLE 5:** Results of the multinomial logistic regression model for people taking one psychotropic drug

Variables	*B* (Se)	Wald *χ*2	*Df*	Sig.	95% CI for odds ratio		
					Inferior	Odds ratio	Superior
Taking one psychotropic drug							
Age	−0.022 (0.016)	2.051	1	.152	0.949	0.978	1.008
*Gender*							
Female (reference)							
Male	0.028 (0.221)	.017	1	.898	0.667	1.029	1.586
*Severity of intellectual disability*							
Mild	0.240 (0.321)	.561	1	.544	0.678	1.272	2.385
Moderate	0.207 (0.273)	.574	1	.449	0.720	1.229	2.098
Severe/profound (reference)							
*Living environment*							
Residential setting	0.736 (0.235)	9.784	1	.002*	1.316	2.087	3.310
Community setting (reference)							
*Communication type*							
None	−1.043 (1.203)	.752	1	.386	0.033	0.352	3.721
Verbal	−0.221 (0.352)	.394	1	.530	0.402	0.802	1.598
Non‐verbal (reference)							
*Mental health conditions*							
No (reference)							
Yes	−2.752 (0.281)	95.773	1	.000*	0.037	0.064	0.111
*Behavioural problems*							
No (reference)							
Yes	−0.136 (0.238)	3.327	1	.568	0.547	0.873	1.392

**TABLE 6:** Results of the multinomial logistic regression model for people taking two or more psychotropic drugs

Taking two or more psychotropic drugs (polypharmacy)							
Age	−0.036 (0.014)	7.124	1	0.008*	0.939	0.964	0.990
*Gender*							
Female (reference)							
Male	0.212 (0.191)	1.233	1	0.267	0.850	1.237	1.800
*Severity of intellectual disability*							
Mild	0.689 (0.273)	6.372	1	0.012*	1.166	1.991	3.399
Moderate	0.272 (0.238)	1.312	1	0.252	0.824	1.313	2.092
Severe/profound (reference)							
*Living environment*							
Residential setting	1.015 (0.205)	24.580	1	0.000*	1.847	2.759	4.120
Community setting (reference)							
*Communication type*							
None	−1.596 (1.219)	1.713	1	0.191	0.019	0.203	2.212
Verbal	−0.026 (0.316)	.007	1	0.935	0.552	1.026	1.906
Non‐verbal (reference)							
*Mental health conditions*							
No (reference)							
Yes	−3.310 (0.266)	154.756	1	0.000*	0.022	0.037	0.062
*Behavioural problem*							
No (reference)							
Yes	−0.822 (0.216)	14.542	1	0.000*	0.288	0.439	0.671

*Note*: *R* ^2^ = 0.38 (Cox & Snell); 0.45 (Nagelkerke). Model *χ* ^2^(18, 991) = 469.80; *p* < .001.

* Significant differences *p* < .05.

## 4 DISCUSSION

The first objective of this study was to describe the mental health and behavioural problems exhibited by people with intellectual disability over the age of 45 in Spain, as well as the type of psychotropic medication they use. Destructive disorders, anxiety disorders, depressive disorders and schizophrenia spectrum disorders were the most prevalent mental health conditions in this population group. The most frequently reported behavioural problems were stereotyped behaviour, aggressive behaviour, and disruptive behaviour. Regarding psychotropic drugs, 38.4% of the sample used any psychotropic drug. This result is in line with the data described by Bowring et al. (2017), and Holden and Gitlesen (2004), who respectively reported that 37.7% and 37.4% of their samples used psychotropics.

Antipsychotics were the most frequently used psychotropic medication, which is consistent with the results of previous studies both in adults with intellectual disability (Bowring et al., 2017; Lunsky & Modi, 2018; Perry et al., 2018; Scheifes, Egberts, et al., 2016; Song et al., 2020) and in older adults with intellectual disability (O'Dwyer et al., 2017). The intake of anxiolytics, antidepressants and antipsychotics was greater among individuals with destructive disorders, stereotyped behaviour, disruptive behaviour, and aggressive behaviour. This result is consistent with previous studies where psychiatric medication is higher in individuals who exhibit more severe challenging behaviours (Deb et al., 2015). The reason for this greater use could be that public health professionals are not adequately trained to properly address the behavioural issues of people with intellectual disability and may use psychotropic medication instead of other strategies (e.g., positive behavioural support), since they lack the necessary understanding of chemical restraint, its side effects, and alternatives (Donley et al., 2012). Another possibility is that certain behavioural problems could be misinterpreted as psychiatric disorders rather than as responses of the person with intellectual disability to certain environmental or personal stressors, especially in those with communication problems (Axmon, Ahlström, et al., 2019; Gomes et al., 2019; Perry et al., 2018; Tsiouris et al., 2013).

The second aim of the study was to analyse the extent to which certain variables predict the intake of psychotropic medication in older people with intellectual disability. Older age, mild intellectual disability, residential settings, and having mental health and behavioural problems contribute to a greater intake of psychotropic medication among aging people with intellectual disability. Specifically, our findings suggest that as the participants' age increases, so does the probability of using more psychotropic medication, as already noted by Bowring et al. (2017). This increase could be due to difficulty in identifying the onset of age‐associated diseases such as dementia, whose prevalence in older adults with intellectual disability is higher than in older adults without intellectual disability (Cooper et al., 2018). Likewise, emotional stress associated with the transitions at this stage of life could be triggering behavioural disorders that are addressed through psychopharmacological therapy (Deutsch & Burket, 2021; Kelly & Su, 2015).

As for the severity of intellectual disability, the participants with mild intellectual disability showed twice as many probabilities of using more than one psychotropic medication than people whose intellectual disability was more severe, although there are authors who claim that the severity of intellectual disability does not predict the use of psychotropic medication (Bratek et al., 2017; Holden & Gitlesen, 2004; Tsiouris et al., 2013).

Regarding living environment, our results suggest that living in residential settings is associated with a greater intake of psychotropic drugs, which is consistent with the findings of other studies both on adults (Kelly & Su, 2015; Lunsky & Modi, 2018) and on individuals who are aging (Bratek et al., 2017; O'Dwyer et al., 2017). This result may be due in part to the characteristics of residential environments (e.g., overcrowding may increase the occurrence of challenging behaviours by some users; McGillivray & McCabe, 2005). On the other hand, direct support professionals in these settings assist a large number of people, and when problematic behaviours appear, they might use restrictive practices (Saloviita, 2002) such as chemical restraint, as opposed to more individualised ones such as positive behavioural support.

In this study, the probability of using two or more drugs was higher among individuals with mental health conditions than in the rest of the participants. This result is consistent with other studies on older adults with intellectual disability, such as those by Chitty et al. (2016) and O'Dwyer et al. (2016). Albeit certain cases require psychotropic medication as the first choice of treatment for mental issues, medication should only be used to alleviate the person's distress, improving their functioning, and fostering their inclusion in social and family life, ultimately improving their quality of life (Novell et al., 2005).

Finally, behavioural problems also seemed relevant in psychotropic medication intake among aging individuals with intellectual disability. According to our results, the probability of polypharmacy was almost three times greater when the assessed individuals exhibited some type of behavioural issue. This is in line with other findings where behavioural problems were associated with starting psychotropic medication, in general (Song et al., 2020) and antipsychotics, in particular (de Kuijper et al., 2010). It would be interesting for future lines of research to investigate whether behavioural problems also predict the intake of one psychotropic drug and not only two or more as concluded in our study. This will shed more light on the relationship between behavioural problems and psychotropic drugs intake.

Because of the negative impact that the side effects of psychotropic medication have on the person's quality of life, it is not the most advisable treatment to address behavioural problems (Scheifes, Walraven, et al., 2016). Moreover, medication side effects themselves may contribute to the development of challenging behaviours, making it necessary to reduce doses, discontinue the drug, or eliminate polypharmacy (Deutsch & Burket, 2021). Psychotropic medication should be reduced, as suggested in the NICE Guidelines (2015) guidelines, to challenging behaviours that do not respond to alternative interventions or that might involve a serious threat to the person or others.

This study is, to date, the only one in Spain to provide a detailed analysis of the use of medication in people with intellectual disability over the age of 45. The sample size allows for estimates with high statistical power. Nonetheless, this work has certain limitations that should be considered. First, the sample used was selected among people who attended third‐sector organisations, which makes it a convenience sample. According to some authors, the psychotropic prevalence estimates in studies where this type of clinical samples is used tend to be quite high (Bowring et al., 2017). Second, this study does not include older population without intellectual disability among its participants, which precludes an analysis of the extent to which both the prevalence of mental health and behavioural problems and psychotropic drug consumption differ between these two groups. Third, the doses of psychotropic medication were not recorded in all cases due to missing information. It would be interesting to take this into account in future studies to determine how to achieve the desired effect with the lowest possible dose when medication is needed, thus minimising the onset of adverse effects associated with these psychotropic drugs. Finally, and although the research team was available to resolve any doubts or setbacks that could arise during the data collection process, given the exploratory nature of the study we relied on proxy reports due to the difficulties associated with reviewing medical/behavioural records for all participants. Also, coding of the psychotropic drugs consumed by the participants was carried out by a single researcher, so no data on inter‐rater reliability can be provided. Future research studies should aim to overcome this limitation by reviewing the medical/psychiatric history of the participants, applying on‐site standardised behavioural assessment instruments, and creating a medication record sheet that can be filled out by the person's referring physician, indicating dosage.

Future research lines should also elaborate on how to address behavioural problems in aging people with intellectual disability using nonpharmacological intervention alternatives (e.g., positive behavioural support, active support) to determine their effectiveness and prioritise their choice in cases of challenging behaviours that respond adequately to this type of interventions.

## 5 CONCLUSION

This study analysed the pattern of psychotropic drug intake in 991 aging individuals with intellectual disability. The results obtained suggest that the greatest use of anxiolytics, antidepressants and antipsychotics was made by individuals with destructive disorders, stereotyped behaviour, oppositional and/or aggressive behaviour. Older age, mild intellectual disability, living in residential facilities, mental health conditions and behavioural problems contribute to increase the amount of psychotropic medication among aging people with intellectual disability.

## Data Availability

The data that support the findings of this study are available from the corresponding author (Patricia Navas), upon reasonable request.
